# Evaluation of *ERBB2* mRNA Expression in HER2-Equivocal (2+) Immunohistochemistry Cases

**DOI:** 10.3390/cancers15061688

**Published:** 2023-03-09

**Authors:** Irene Carretero-Barrio, Tamara Caniego-Casas, Marta Rosas, María Concepción Sánchez, Noelia Martínez-Jáñez, Miguel Chiva, David Sarrió, Gema Moreno-Bueno, José Palacios, Belén Pérez-Mies

**Affiliations:** 1 Servicio de Anatomía Patológica, Hospital Universitario Ramón y Cajal, Instituto Ramón y Cajal de Investigación Sanitaria (IRYCIS), 28034 Madrid, Spain; irene.carretero@salud.madrid.org (I.C.-B.); tamara.caniego@salud.madrid.org (T.C.-C.); martagrosas@gmail.com (M.R.); 2 Faculty of Medicine, Universidad de Alcalá, 28801 Alcalá de Henares, Spain; 3 Centro de Investigación Biomédica en Red de Cáncer (CIBERONC), 28029 Madrid, Spain; dsarrio@iib.uam.es (D.S.); gmoreno@iib.uam.es (G.M.-B.); 4 Unidad de Patología Mamaria, Hospital Universitario Ramón y Cajal, 28034 Madrid, Spain; mariaconcepcion.sanchez.martinez@salud.madrid.org (M.C.S.); nmartinezj@salud.madrid.org (N.M.-J.); miguel.chiva@salud.madrid.org (M.C.); 5 Servicio de Ginecología, Hospital Universitario Ramón y Cajal, Instituto Ramón y Cajal de Investigación Sanitaria (IRYCIS), 28034 Madrid, Spain; 6 Servicio de Oncología, Hospital Universitario Ramón y Cajal, Instituto Ramón y Cajal de Investigación Sanitaria (IRYCIS), 28034 Madrid, Spain; 7 Servicio de Radiología, Hospital Universitario Ramón y Cajal, Instituto Ramón y Cajal de Investigación Sanitaria (IRYCIS), 28034 Madrid, Spain; 8 Departamento de Bioquímica, Universidad Autónoma de Madrid (UAM), Instituto de Investigaciones Biomédicas ‘Alberto Sols’, Conexión Cáncer (UAM-CSIC), 28029 Madrid, Spain; 9 Fundación MD Anderson Internacional, 28033 Madrid, Spain

**Keywords:** breast carcinoma, HER2, mRNA, immunohistochemistry

## Abstract

**Simple Summary:**

HER2-equivocal cases represent around 15% of breast carcinomas, and 20–40% of them are HER2-amplified. The distinction between HER2-amplified and non-amplified cases is of great importance for patient management, and in this study, we investigated the performance of STRAT4 (a RT-qPCR platform) in the evaluation of HER2-equivocal cases. We compared this technique to the recommended methods (immunohistochemistry and in situ hybridization) for the evaluation of HER2-equivocal cases and non-equivocal HER2 cases. We found a 91.3% accuracy for the identification of HER2-positive tumors globally and 99.3% for that of non-equivocal HER2 cases, while the accuracy decreased to 80.7% for HER2-equivocal cases. Our results suggest that STRAT4 is not reliable for the evaluation of the HER2 amplification status in equivocal cases.

**Abstract:**

Xpert Breast Cancer STRAT4 is a RT-qPCR platform that studies the mRNA expression of *ESR1*, *PGR*, *MKI67* and *ERBB2*, providing a positive or negative result for each of these breast cancer biomarkers. Its concordance with immunohistochemistry (IHC) and in situ hybridization (ISH) has been previously demonstrated, but none of the previous works was focused on HER2-equivocal (2+) cases identified by IHC. Thus, we studied the concordance between IHC/ISH and STRAT4 results for 112 HER2 2+ IBC samples, using 148 HER2 0+, 1+ and 3+ (no-HER2 2+) samples for comparison. We found 91.3% accuracy for the determination of HER2 status globally, 99.3% for no-HER2 2+ samples and 80.7% for HER2 2+ samples. Regarding the other biomarkers, we obtained 96.4% accuracy for estrogen receptor, 84.1% for progesterone receptor and 58.2% for Ki67. Our results suggest that the use of *ERBB2* mRNA for the evaluation of HER2 2+ cases is not a reliable reflex method to assess the *ERBB2* amplification status.

## 1. Introduction

The assessment of biomarkers expression in invasive breast carcinoma (IBC) is crucial for patient management. Currently, it is performed by evaluating the expression of the estrogen receptor (ER), progesterone receptor (PR), Ki67 and HER2. The expression of ER and PR is present in about 75% to 80% of IBC [1], especially in well and moderately differentiated tumors, and these patients are eligible for endocrine therapy. The recommended method for their evaluation is immunohistochemistry (IHC), assessing the percentage of positive tumoral cells and the staining intensity [1].

Ki67 is widely used in pathology to evaluate cell proliferation rate. In IBC, it is used as a prognostic factor and for treatment planning [2]. However, it is not widely accepted in clinical practice due to its variability because of preanalytical factors and low intra- and interobserver reproducibility, leading to different attempts to standardize its evaluation [3].

HER2 is overexpressed in approximately 15–20% of IBC, and only these tumors are eligible for anti-HER2 therapy. Its evaluation is usually performed with IHC, scoring the tumors as negative (score 0 or 1+), equivocal (score 2+) and positive (score 3+). In cases that show an equivocal HER2 immunohistochemical pattern (HER2 2+, according to 2018 ASCO-CAP guidelines [4]), additional testing must be performed using in situ hybridization (ISH) to determine its final positive (amplified) or negative (no amplified) status. Moreover, in some cases, unusual patterns of HER2 might be encountered, which also warrant further testing [5]. HER2 2+ represent around 15% of IBC [6], and HER2 amplification is detected in 20–40% of them [7,8,9]. 

Although it has been proven that patients with HER2 1+ and HER2 2+ tumors without amplification do not benefit from adjuvant trastuzumab therapy [10], recent studies have shown that the outcome of these patients may improve with HER2-directed antibody–drug conjugates [11]. Thus, the distinction between HER2 0+ and HER2 1+ is gaining relevance, as is the concept of “HER2-low” breast cancer, encompassing those HER2 1+ and HER2 2+ tumors without HER2 amplification [5,12].

As previously mentioned, HER2 2+ cases need additional testing with ISH, and this technique requires additional tissue and takes at least 2 days to be performed. However, new technologies are becoming available to assess IBC biomarkers. In this regard, Xpert® Breast Cancer STRAT4 mRNA (Cepheid, Sunnyvale, CA, USA) (STRAT4) is a real-time quantitative polymerase chain reaction (RT-qPCR) assay that studies the expression of *ESR1*, *PGR*, *ERBB2* and *MKI67* mRNA, providing a positive or negative result for each biomarker in approximately two hours [13]. Other advantages of this technique are its reproducibility and objectivity, as the software provides these results.

Previous studies reported the concordance of STRAT4 and immunohistochemistry for ER, PR, Ki67 and HER2, achieving good results [13,14,15,16,17,18,19]. We previously collaborated in a Europe-wide external quality assessment [14], achieving accuracies ≥90% for all biomarkers across five participating centers, highlighting the reproducibility of this technique. However, none of these STRAT4 studies were focused on the complex and salient group that comprises HER2 2+ cases. 

Thus, the aim of this study was to investigate the performance of STRAT4 in the evaluation of HER2 2+ cases, using FISH amplification status as the gold standard for the final HER2 classification.

## 2. Materials and Methods

### 2.1. Case Selection

Consecutive HER2 2+ IBC samples diagnosed in our department between the years 2012 and 2018 were selected, all of them from female patients, for which there was enough tissue to perform the mRNA assay (see Section 2.3). In addition, consecutive HER2 0 + , 1+ and 3+ cases (from now on, these will be referred as no-HER2 2+ cases) diagnosed in our department between 2012 and 2018 were selected for comparison. This study was approved by the Ethics Committee of the Hospital Universitario Ramón y Cajal (reference number 361-20).

### 2.2. Immunohistochemistry

The antibodies used are shown in Table 1. A positive external control was placed on each immunohistochemical slide. The results were interpreted according to the last ASCO-CAP guidelines: 2020 ASCO-CAP guidelines [1] for ER and PR; and 2018 ASCO-CAP guidelines [4] for HER2. Ki67 was evaluated following the Updated Recommendations from the International Ki67 in Breast Cancer Working Group [3] and it was considered as indicative of a high-proliferative state when >20% of tumoral cells showed nuclear staining, as reported in previous studies of STRAT4 [13,14,17].

All HER2 IHC slides were reviewed by two pathologists, and discordances were resolved by consensus. Additional HER2 testing was performed in HER2 2+ cases, defined as weak to moderate complete membrane staining observed in >10% of tumor cells [4]. HER2 fluorescent in situ hybridization (FISH) was performed for them, using the PathVysion HER-2 DNA Probe Kit (Abbot Laboratories, Chicago, IL, USA). The results were interpreted following the 2018 ASCO-CAP guidelines [4].

### 2.3. mRNA Expression with Xpert^®^ Breast Cancer STRAT4

The samples were processed according to the manufacturers´ instructions, requiring at least 30% of cellularity. Briefly, each specimen was cut three times obtaining 4 μm samples that were placed in a 1.5 mL Eppendorf tube. This material was then mixed with 1.2 mL of lysis reagent and 20 µL of proteinase K. Subsequently, it was incubated at 80 °C for 30 min and mixed with 1.2 mL of ethanol ≥95%. For each sample, 520 µL of the solution was transferred to the sample chamber of a STRAT4 cartridge and placed into a GeneXpert module for RNA extraction, purification and RT-qPCR analysis.

The results were analyzed using the GeneXpert DX software. *CYFIP1* was used as a reference for mRNA expression, and a delta cycle threshold (dCt = [Ct*_CYFIP1_*] − [Ct_target_]) was provided that uses a predefined cut-off value to classify the expression status of each biomarker (positive vs negative). In the case of *ERBB2*, the cut-off value is set to −1, for *ESR1* to −1, for *PGR* to −3.5, and for *MKI67* to −4 [13]. Thus, the expression of *CYFIP1* was used both to normalize the biomarkers expression and as an mRNA quality control of the sample.

### 2.4. Statistical Analysis

All analyses were performed using R 4.1.0 [20]. The diagnosis accuracy (sensitivity, specificity, positive predictive value and negative predictive value) was studied. Comparisons of the means were performed using Wilcoxon signed-rank test, and correlation studies using the Spearman coefficient. The results were considered statistically significant if *p* < 0.05.

## 3. Results

### 3.1. Clinicopathological Features

A total of 260 cases were studied from 256 females (three multifocal and one bilateral), being 112 cases HER2 2+. The clinical and histopathological features are summarized in Table 2. Briefly, the median patient age was 58.9 years, 55.4% of the cases presented a histological grade 2, and most of them (81.2%) were IBC of no special type. Most expressed hormonal receptors (80.8% ER and 63.8% PR), 43% were HER2 2+, and 66.9% had a low-proliferative index.

### 3.2. STRAT4 Analysis

A summary of the STRAT4 results is shown in Table 3. Most cases were hormonal receptor-positive (80.4% for *ESR1* and 73.5% for *PGR*), 73.5% were *ERBB2*-negative, and 27.3% rendered a negative *MKI67* result. The histopathological features of the cases with a failed STRAT4 determination (*n* = 8, 3.2%) are shown in Table S1.

### 3.3. Diagnostic Accuracy Analysis

The eight cases with a failed STRAT4 determination were excluded in the subsequent analysis. The number of concordant and discordant cases for the different biomarkers is shown in Table 4 and a summary of the diagnostic accuracy statistics is shown in Table 5. The accuracy for HER2 determination reached 91.3% when studying all cases together, while it decreased to 80.7% for HER2 2+ cases and increased to 99.3% for no-HER2 2+ cases. The biomarkers results with both techniques of the discordant cases are shown in Table S2.

### 3.4. HER2 Results

**Figure 1:**
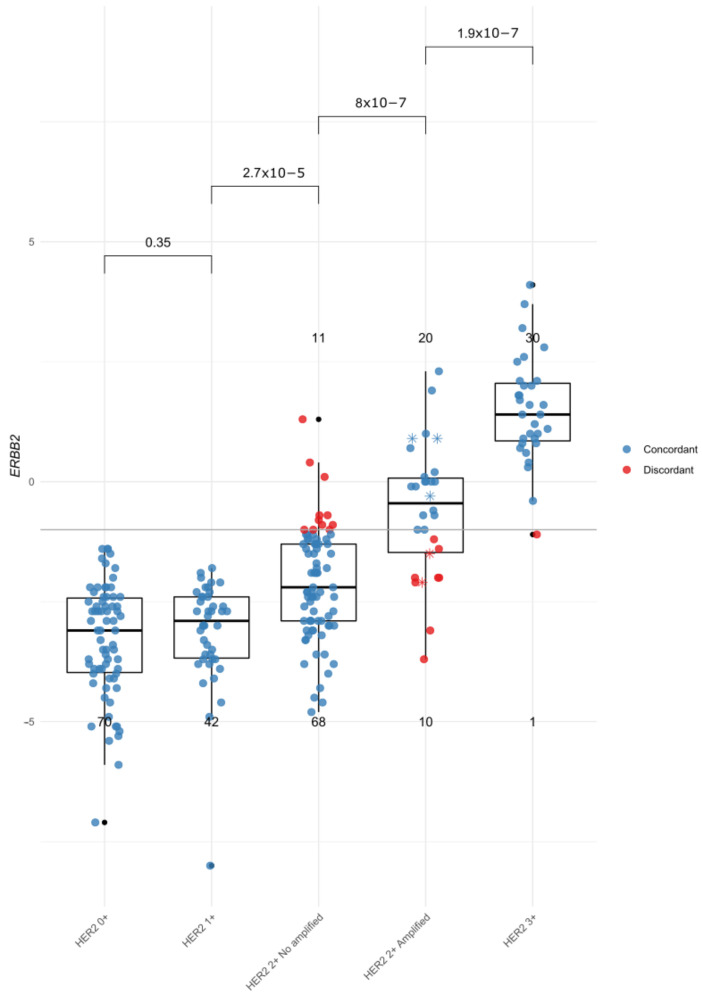
Boxplots of STRAT4 *ERBB2* dCt by HER2 immunohistochemical (0+, 1+, 2+, 3+) and FISH (not amplified, amplified) results. Each individual case is represented with a colored point (blue for concordant cases, red for discordant cases). Asterisks (*) in the HER2 2+-amplified group represent the cases with signals amplified in clusters (*n* = 5). The horizontal gray line represents the cut-off value for STRAT4 *ERBB2* dCt (−1). The bars above the boxplots represent the *p*-values of *ERBB2* dCt comparison of the means.

The STRAT4 *ERBB2* dCt for each case according to their immunohistochemical and FISH HER2 status is shown in Figure 1. Twenty-one out of the 109 HER2 2+ cases (19.3%) and 1 out of the 31 HER2 3+ cases (3.2%) showed discordant results between mRNA expression and FISH amplification. All HER2 0+ and HER2 1+ cases where correctly categorized as HER2-negative by STRAT4. There were statistically significant differences between the *ERBB2* dCt and the IHC/FISH results among all subgroups, except between HER2 0+ and HER2 1+.

Regarding the HER2 2+ cases without HER2 amplification analyzed by FISH, 11 out of 79 (13.9%) were classified as HER2-positive by STRAT4. All of them showed ≤2 HER2/CEP17 ratio and ≤4 mean HER2 copies (group 5, ISH-negative) according to the CAP-ASCO guidelines [4]). Eight of these cases scored near the STRAT4 cut-off for *ERBB2* (dCt −0.7 to −1, being the cut-off for positivity ≥ −1), while three scored far above this point (dCt 0.1, 0.4 and 1.3).

Ten out of thirty (33.3%) HER2 2+ cases with HER2 amplification by FISH were classified as HER2-negative by STRAT4. Two of them showed *ERBB2* signals amplified in clusters, with dCt values of −1.5 and −2.1. The remaining eight discordant cases showed ≥2 HER2/CEP17 ratio and ≥4 mean HER2 copies (group 1, ISH-positive) according to the CAP-ASCO guidelines [4]). As opposed to the non-amplified cases, all but one amplified sample (dCt −1.2) scored far below the cut-off point (dCt −1.4 to −3.7).

**Figure 2:**
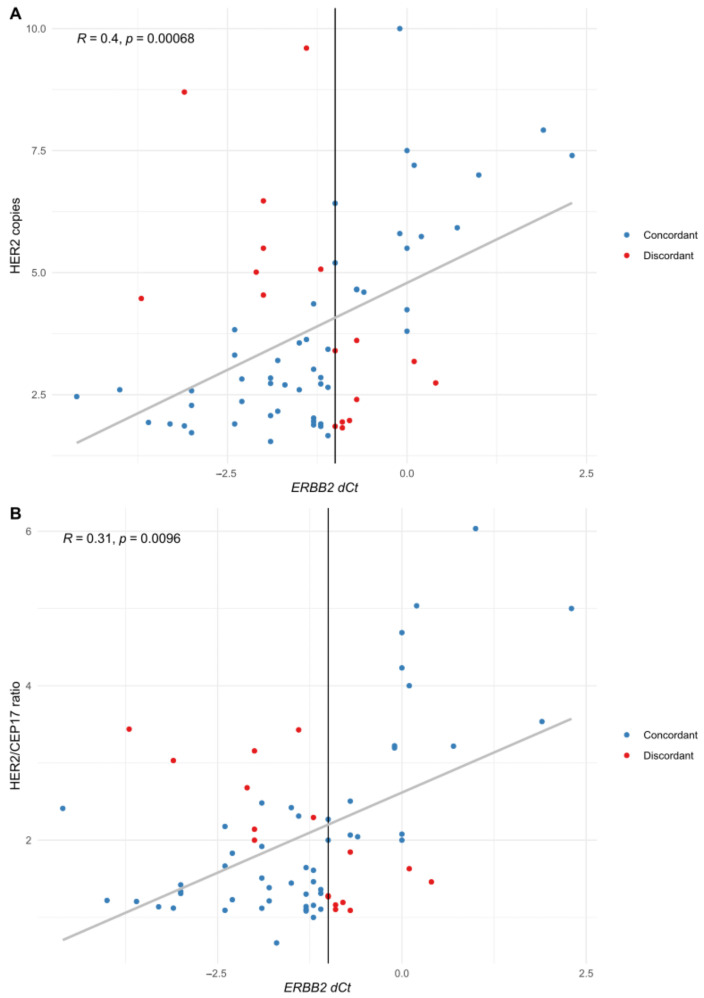
Correlation of HER2 copies (**A**) and HER2/CEP17 ratio (**B**) with *ERBB2* expression. Each individual case is represented with a colored point (blue for concordant cases, red for discordant ones). The vertical line represents the cut-off value for STRAT4 *ERBB2* dCt (−1).

When studying the relationship between STRAT4 dCt and mean HER2 copies and HER2/CEP17 ratio, there was a positive correlation, as shown in Figure 2 (cases with HER2 signals amplified in clusters (*n* = 5) were excluded from these analyses because the number of HER2 signals could not be accurately counted).

**Figure 3:**
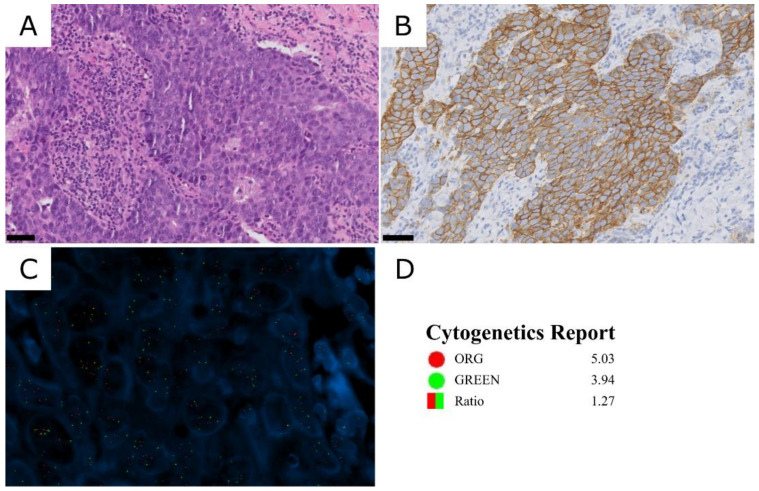
HER2 3+ case classified as HER2 negative by STRAT4 (case 279). (**A**) HE. (**B**) HER2 immunohistochemistry, showing intense and complete membrane staining in >10% of tumor cells. (**C**) HER2 FISH, showing gains for both probes. Blue: DAPI, green signals: CEP17 probe; red signals: ERBB2 probe. (**D**) Cytogenetics report. ORG: *ERBB2* probe count; GREEN: CEP17 probe count. Scale bar: 50 µm.

As illustrated previously, one HER2 3+ case showed discordant results between the HER2 immunohistochemical status and STRAT4, near the cut-off value of *ERBB2* mRNA expression (dCt −1.1) (Figure 1). Due to this discordance, HER2 FISH was performed, which showed gains in both CEP17 and *ERBB2* signals (ISH group 4 [4]) (Figure 3). This patient was treated with neoadjuvant therapy, including pertuzumab, trastuzumab and docetaxel, showing a pathological complete response in the subsequent surgical specimen.

### 3.5. Estrogen Receptor Results

**Figure 4:**
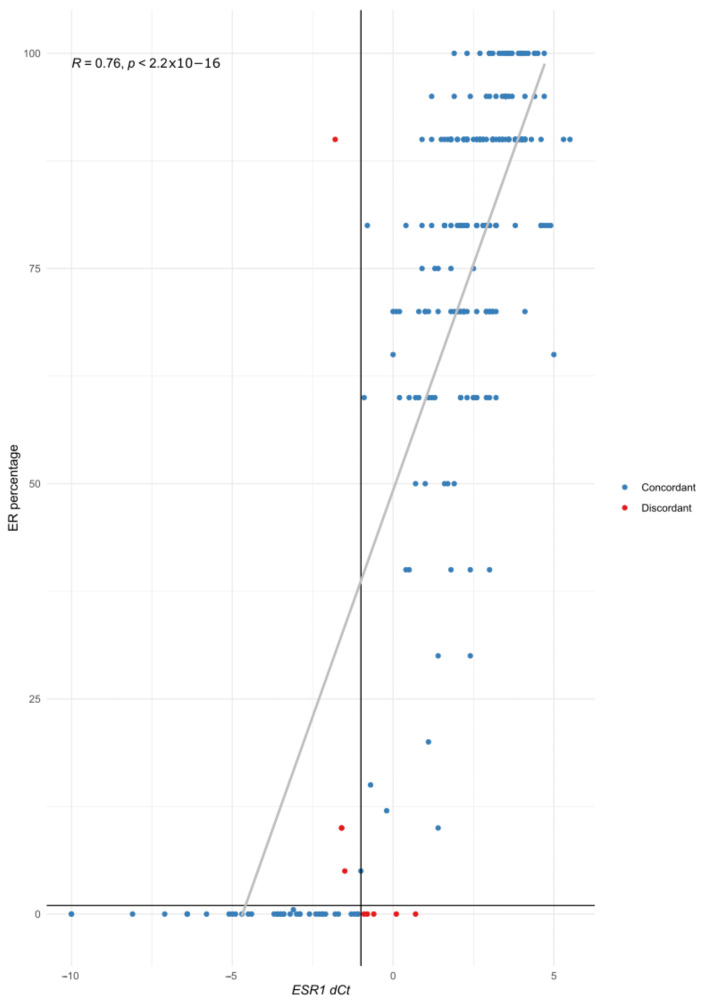
Correlation of ER IHC percentage (*y* axis) with *ESR1* dCT (*x* axis). Each individual case is represented with a colored point (blue for concordant cases, red for discordant ones). The vertical line represents the cut-off value for STRAT4 *ESR1* dCt (−1), and the horizontal line the cut-off value for ER (1%).

When comparing the ER results, nine cases (3.6%) showed discordant results. Three out of four discordant IHC-positive cases showed low ER expression (5% of ER-positive cells (*n* = 1) and 10% of ER-positive cells (*n* = 2)). The IHC ER percentage and *ESR1* dCt showed a positive correlation (Figure 4).

### 3.6. Progesterone Receptor Results

**Figure 5:**
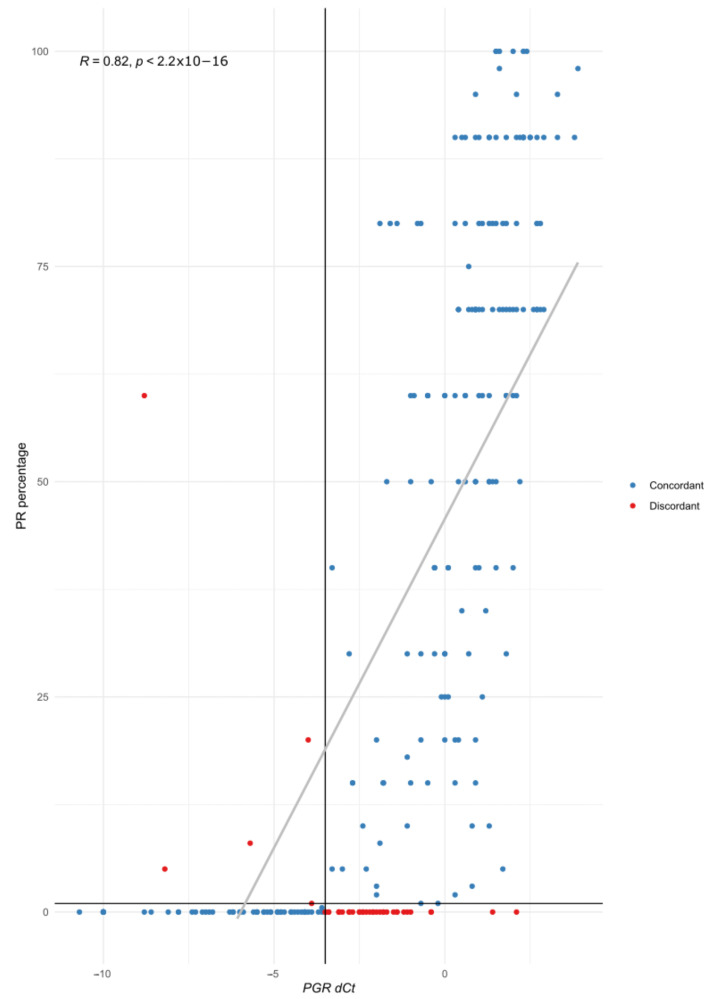
Correlation of PR IHC percentage (*y* axis) with *PGR* dCT (*x* axis). Each individual case is represented with a colored point (blue for concordant cases, red for discordant ones). The vertical line represents the cut-off value for STRAT4 *PGR* dCt (−3.5), and the horizontal line the cut-off value for PR IHC (1%).

Regarding PR, 40 cases (15.9%) showed discordant results, and 35 were IHC-negative and STRAT4-positive. The IHC PR percentage and *PGR* dCt showed a positive correlation (Figure 5).

### 3.7. Ki67 Results

**Figure 6:**
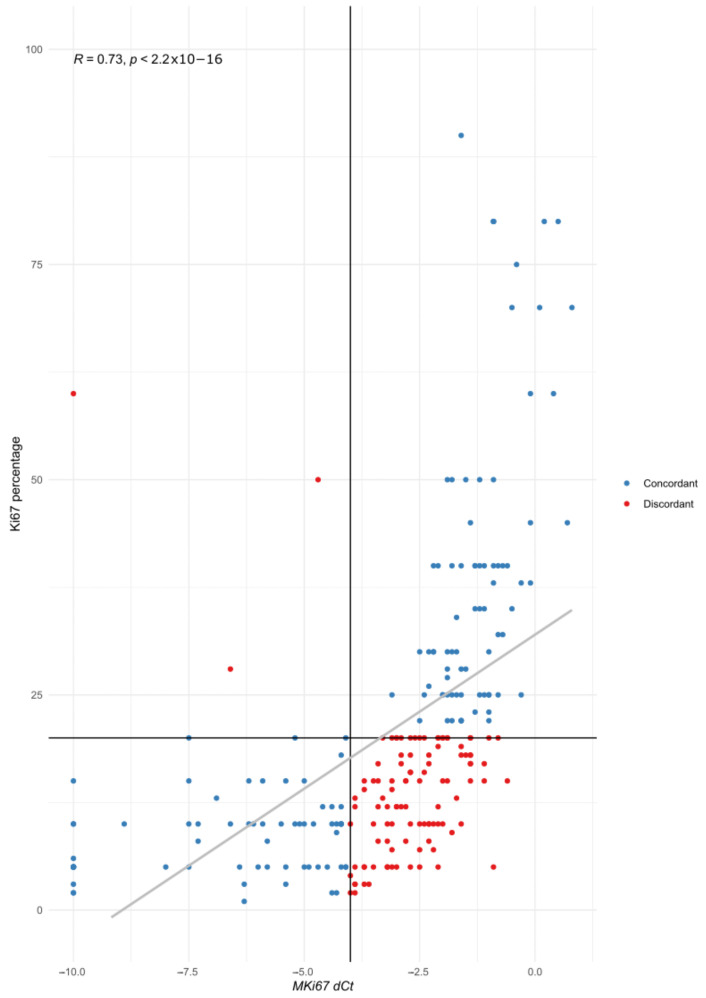
Correlation of Ki67 IHC percentage (*y* axis) with *MKI67* dCT (*x* axis). Each individual case is represented with a colored point (blue for concordant cases, red for discordant ones). The vertical line represents the cut-off value for STRAT4 *MKI67* dCt (−4), and the horizontal line the cut-off value for Ki67 IHC (20%).

One hundred and five cases (41.7%) showed discordant results between IHC and STRAT4. The IHC Ki67 percentage and *MKI67* dCt showed a positive correlation (Figure 6). Discordant cases with Ki67 expression <5% (*n* = 7) showed a STRAT4 dCt near the cut-off value (range −3.6 to −4).

## 4. Discussion

In this study, we evaluated the performance of STRAT4, a RT-qPCR platform, to assess breast cancer biomarker status in a cohort enriched in HER2 2+ cases. An acceptable concordance was observed between IHC and STRAT4 when evaluating HER2 globally and no-HER2 2+ cases. However, the concordance clearly decreased in HER2 2+ tumors. Thus, STRAT4 achieved a high concordance with IHC in HER2 determination in no-HER 2+ cases, with a 99.3% accuracy. However, HER2 2+ cases still represent a challenge, with an identification accuracy of 80.7%. This degree of discordance was not attributable to the specific series, since 26.8% of our HER2 2+ cases showed HER2 amplification by FISH, in accordance with the literature [7,8,9]. In addition, all cases rendered interpretable results for other STRAT4 markers (Supplementary Table S2), and the failure rate of STRAT4 (3.2%) in this series was similar to that reported in the literature [13,16,17]. 

Previous studies [13,14,15,17,18,19] reported around 90% accuracy for STRAT4, when studying globally the HER2 status (positive vs. negative), without distinguishing the HER2 2+ amplification status. A more recent study also reported a 90% concordance between the HER2 2+ FISH and STRAT4 results, although only 10 HER2 2+ cases were included [16], and the 2013 ASCO/CAP guidelines were followed [21]. 

The fact that two HER2 2+ cases that showed HER2 amplification with cluster signals were categorized as HER2-negative by STRAT4 is interesting. In these cases, we would expect to find high *ERBB2* mRNA levels and HER2 protein levels, but the dCt value obtained was not near the positivity cut-off, and HER2 IHC was equivocal. Thus, this could be due to a transcription mechanism resulting in low mRNA and protein levels, even in the presence of gene amplification. In fact, several mechanisms play a role in regulating the mRNA and protein levels, such as transcription factors, promoters, micro-RNA or DNA methylation.

The discordant HER2 3+ case might be explained by the genetic alterations found, as we observed a high number of copies of both CEP17 and *ERBB2* with the FISH assay. A polysomy affecting both chromosome 15 (reference gene *CYPIP1*) and 17 (*ERBB2*) could result in higher mRNA levels of both, thus resulting in a low dCt value. Nonetheless, it was suggested that increases in the number of copies of both *ERBB2* and *CEP17* are usually due to pericentromeric gains rather than to polysomies [22]. Downs-Kelly et al. [23] studied the effect of polysomy 17 on HER2 gene and protein expression and found that polysomy does not significantly contribute to a higher expression, although it has been reported that most polysomy cases that show 3+ by IHC are also amplified, as shown by FISH [24]. The FISH result in this discordant case, with a ERBB2/CEP17 ratio <2 and ≥4 and <6 *ERBB2* signals/cell, would indicate it belonged to the ISH group 4. However, following the current ASCO/CAP guidelines [4], this sample should be categorized as HER2-positive, as the initial immunohistochemistry rendered a 3+ status (Figure 3) and in clinical practice it would not have been studied by FISH. In addition, the patient showed a great response to targeted therapy, confirming the HER2-positive status. 

When the aforementioned ASCO/CAP 2013 guidelines [21] were applied, a poorer concordance between STRAT4 and FISH results was obtained, as six STRAT4-negative and non-amplified by FISH tumors (concordant cases using 2018 criteria) would have been classified as HER2-positive by the 2013 guidelines. In this regard, previous studies comparing both guidelines showed that following the 2018 criteria reduces the rate of HER2 positivity [25]. Moreover, it has been proven that the 2018 ASCO/CAP guidelines for HER2 [4] have a better concordance with HER2 mRNA expression assays [26].

There were statistically significant differences in *ERBB2* dCt according to the HER2 status for all groups except the HER2 0+ compared to the HER2 1+ (Figure 1). Due to the recent development of HER2-directed antibody–drug conjugates [11], the correct classification of these cases is gaining importance, as it may change the clinical management of the patients. In this regard, a recent study by Atallah et al. [27] suggests new refined score patterns to distinguish HER2 0+ and HER2 1+ tumors, which correlates with mRNA expression.

Regarding the hormonal receptors, STRAT4 achieved the greatest accuracy in ER determination, in accordance with the literature [13,14,15,17], while for PR had worse results. Although it could be argued that some discrepancies could be attributed to the antibodies used, as they present different sensitivity and specificity, it has been shown that the Agilent/Dako platform has one of the strongest inter-observer agreement and accuracy compared to Leica and Ventana [28]. However, most studies that compared STRAT4 to IHC used Agilent/Dako, with accuracies ranging from 100% to 93.3% for ER (ours was 96.4%) and 89% to 96% for PR (ours was 84.1%) [14,15,16]. Only one study used Ventana, achieving 98.9% accuracy for ER and 89.8% for PR [17]. Another study reported 98.3% accuracy for ER and 86.7% for PR, without specifying the antibodies used [18]. 

Patients with higher ER and PR expression have a better response to endocrine therapy, and even those with as little as 1% positive staining still show a response [1]. Nevertheless, low ER expression (1% to 10% of weakly positive cells) must be reported, as recommended by the current guidelines [1], as these tumors are heterogenous, some of them with similar characteristics to ER-negative carcinomas [29,30], and do not benefit as much from endocrine treatments. Our study did not have sufficient power to evaluate low-ER tumors, and only five cases were classified in this group. However, three of them showed discordant results, highlighting the difficulty of assessing bordering values.

As shown before, previous studies also found a lower accuracy for PR when compared to ER [13,14,15,17,18], and these results are similar to ours. This may be explained by the heterogeneity of PR expression, as it is not as uniform as ER and usually presents a lower intensity, making its evaluation more difficult.

Ki67 expression led to the greatest discordance between the two techniques. In fact, it has the lowest concordance rates between all IHC biomarkers due to the difficulties in the standardization of its measurement [3]. Moreover, previous studies of the concordance of IHC and STRAT4 also found that Ki67 has the lowest accuracy, ranging from 73% to 90% [13,14,17]. However, we obtained even worse results, with 58.2% accuracy. This may be due to methodological differences, as in some studies, cases with intermediate IHC results were excluded from the analysis [17], or a small number of cases were analyzed [14]. There was significant scattering of the *MKI67* dCt data for tumors with low proliferative rates, with Ki67 < 20% (Figure 6), in accordance with previous observations [13,15,17]. The best thresholds for Ki67 IHC and *MKI67* dCt in our cohort according to the ROC curves were 15.5% and −3 dCt, respectively (Figures S1 and S2, versus 20% and −4 dCt used) [31].

The main limitation of our study is that the number of cases was low compared to those of other STRAT4 concordance studies [13,17].

## 5. Conclusions

Although the number of cases we studied was limited, we were able to analyze a large number of HER2 2+ cases, showing that they represent a complex group in which *ERBB2* mRNA is not a reliable reflex method to assess *ERBB* amplification status, although it could be useful in areas where IHC and FISH are not available [16]. 

Further studies should evaluate the concordance of STRAT4 in other special IBC cases, such as those with *ERBB2* mutations as well as HER2-low and low-ER cases, as it could provide useful information on biomarker status and patient management.

## Figures and Tables

**Table 1:** Details of the antibodies used.

Biomarker	Antibody	Company	Concentration
ER	EP1 clone	Agilent	Ready to use
PR	PgR clone	Agilent	1:50
HER2	HercepTest	Agilent	Ready to use
Ki67	Mib-1	Agilent	Ready to use

**Table 2:** Histopathological features of the series.

		Total	Percentage
		260	100
Age, median (IQR)	58.9 (15.9)	256	
Histological grade	1	48	18.5
	2	144	55.4
	3	68	26.2
Histological type	IBC, no special type	211	81.2
	Lobular carcinoma	32	12.3
	Other	17	6.5
ER	Positive	210	80.8
	Low	5	1.9
	Negative	45	17.3
PR	Positive	166	63.8
	Negative	94	36.2
HER2	Positive (3+)	31	11.9
	Equivocal 2+ (amplified)	30	11.5
	Equivocal 2+ (no amplified)	82	31.5
	Negative (1+)	46	17.7
	Negative (0+)	71	27.3
Ki67 ^1^	High-proliferative	86	33.1
	Low-proliferative	173	66.9

^1^ In one no-HER2 2+ case Ki67 immunohistochemical status could not be retrieved.

**Table 3:** STRAT4 results of the series.

		Total	Percentage
ER	Positive	209	80.4
	Negative	43	16.5
	Fail	8	3.1
PR	Positive	191	73.5
	Negative	61	73.5
	Fail	8	3.1
HER2	Positive	61	23.5
	Negative	191	73.5
	Fail	8	3.1
Ki67	Positive	181	69.6
	Negative	71	27.3
	Fail	8	3.1

**Table 4:** Number of concordant and discordant cases for the different biomarkers’ status.

Biomarker	Group	Concordat		Discordant		Total
		Positive	Negative	IHC Positive	IHC Negative	
HER2	Globally	50	180	11	11	252
	HER2 2+	20	68	10	11	109
	No-HER2 2+	30	111	1	0	143
	* HER2 0+ *	0	70	0	0	70
	* HER2 1+ *	0	42	0	0	42
	* HER2 3+ *	30	0	1	0	31
ER		204	39	4	5	252
PR		156	56	5	35	252
Ki67		78	68	3	102	251

**Table 5:** Diagnostic accuracy statics for each biomarker status.

Biomarker	Group	Accuracy	Sensitivity	Specificity	Positive Predictive Value	Negative Predictive Value
HER2	Globally	91.3	82.0	94.2	82.0	94.2
	HER2 2+	80.7	66.7	86.1	64.5	87.2
	No-HER2 2+	99.3	96.8	100	100	99.1
	* HER2 0+ *	-	-	100	-	100
	* HER2 1+ *	-	-	100	-	100
	* HER2 3+ *	-	96.8	-	100	-
ER		96.4	98.1	88.6	97.6	90.7
PR		84.1	96.9	61.5	81.7	91.8
Ki67		58.2	96.3	40.0	43.3	95.8

## Data Availability

The datasets used and/or analyzed during the current study are available from the corresponding authors upon reasonable request.

## Supplementary Materials

The following supporting information can be downloaded at: https://www.mdpi.com/article/10.3390/cancers15061688/s1, Table S1: Histopathological features of the cases with a failed STRAT4 determination; Table S2: Biomarkers results of both techniques for the discordant cases; Figure S1: ROC curve for the best *MKI67* dCt threshold in our series. Figure S2: ROC curve for the best Ki67 IHC threshold in our series.
