# Supplementary material for: Evaluation of ERBB2 mRNA Expression in HER2-Equivocal (2+) Immunohistochemistry Cases
Source: Cancers (Basel). 2023 Mar 9;15(6):1688. doi: 10.3390/cancers15061688 (PMC10046044; doi:10.3390/cancers15061688)
Supplement: Supplementary file 1 [file cancers-15-01688-s001.zip › cancers-2211728-supplementary.pdf]

Supplemental Figures

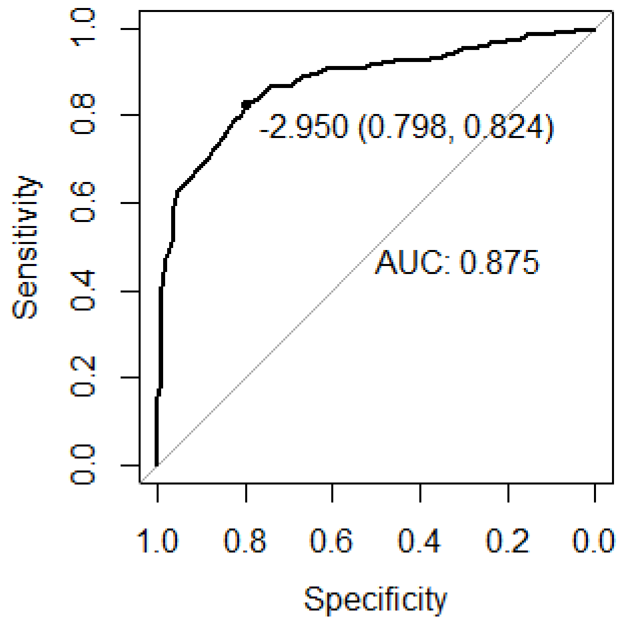

Supplemental Figure S1: ROC curve for the best MKi67 *dCt threshold in our series.*

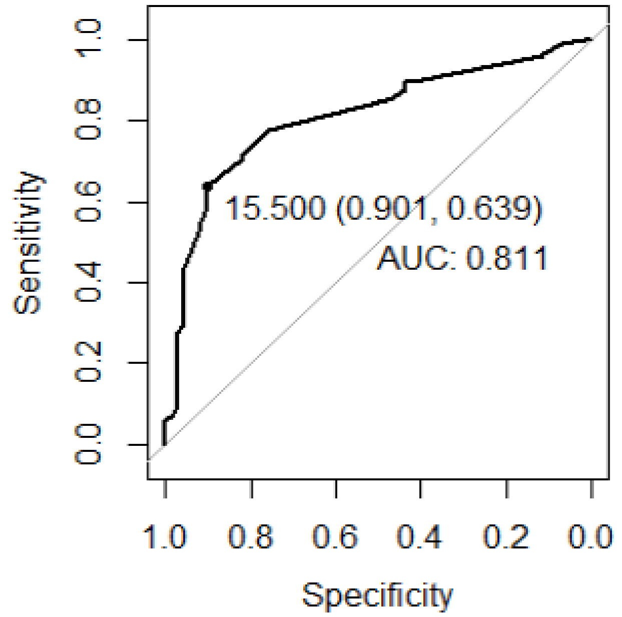

Supplemental Figure S2: ROC curve for the best Ki67 IHQ threshold in our series.

Supplemental Table S1. Histopathological features of the cases with a failed STRAT4 determination.

		Total<br>8	Percentage<br>100
Histological grade	1	1	12.5
	2	3	37.5
	3	4	50
Histological type	IBC, no special type	8	100
ER	Positive	1	12.5
	Negative	7	87.5
PR	Positive	5	62.5
	Negative	3	37.5
HER2	Equivocal 2+ (no amplified)	3	37.5
	Negative (1+)	4	50
	Negative (0+)	1	12.5
Ki67	Positive	2	33.1
	Negative	6	75

Supplemental Table S2. Biomarkers results of both techniques for the discordant cases.

ID	HER2 IHQ	HER2 FISH	ERBB2 dCt	HER2 concordance	ER %	ESR1 dCt	ER concordance	PR %	PGR dCt	PR concordance	Ki67 %	MKi67 dCt	Ki67 concordance	Sample type
2	2	Negative	-1.3	Concordant	70	2.2	Concordant	0	-3.5	Discordant	8	-2.3	Discordant	Core biopsy
4	2	Positive	0	Concordant	90	3.1	Concordant	0	-3	Discordant	14	-3.1	Discordant	Core biopsy
5	2	Positive	-2	Discordant	80	2.1	Concordant	18	-1.1	Concordant	45	-1.4	Concordant	Core biopsy
7	2	Positive	1	Concordant	0	0.1	Discordant	0	-4.5	Concordant	5	-2.1	Discordant	Core biopsy
8	2	Positive	-2.1	Discordant	0	-5.1	Concordant	0	-5.5	Concordant	40	-1.1	Concordant	Core biopsy
11	2	Positive	-3.1	Discordant	0	-0.6	Discordant	0	-2.5	Discordant	18	-4.2	Concordant	Core biopsy
13	2	Positive	0.2	Concordant	80	0.4	Concordant	0	-3.1	Discordant	20	-2.1	Discordant	Core biopsy
16	2	Positive	-1.5	Discordant	100	3	Concordant	0	-2.4	Discordant	16	-2.4	Discordant	Core biopsy
18	2	Positive	-1	Concordant	100	4	Concordant	90	2.1	Concordant	17	-2.9	Discordant	Core biopsy
20	2	Positive	0	Concordant	60	0.7	Concordant	0	-2.3	Discordant	38	-0.9	Concordant	Core biopsy
23	2	Positive	0.1	Concordant	90	3.1	Concordant	15	-1	Concordant	8	-2.8	Discordant	Core biopsy
24	2	Positive	-1.2	Discordant	100	3.3	Concordant	100	2.4	Concordant	25	-1.8	Concordant	Core biopsy
25	2	Positive	-2.1	Discordant	10	-1.6	Discordant	0	-7	Concordant	80	-0.9	Concordant	Core biopsy
28	2	Negative	-0.9	Discordant	100	2.3	Concordant	70	0.8	Concordant	20	-3.1	Discordant	Core biopsy

29	2	Negative	-1.8	Concordant	60	2.9	Concordant	50	2.2	Concordant	18	-1.6	Discordant	Core biopsy
30	2	Negative	-1.2	Concordant	75	0.9	Concordant	90	1	Concordant	10	-2.2	Discordant	Core biopsy
31	2	Negative	-1.1	Concordant	100	1.9	Concordant	100	1.6	Concordant	15	-2.8	Discordant	Core biopsy
32	2	Negative	-3.6	Concordant	0	-0.8	Discordant	0	-2.5	Discordant	80	-0.9	Concordant	Core biopsy
33	2	Negative	-0.8	Discordant	90	2.3	Concordant	5	-3.3	Concordant	10	-2.3	Discordant	Core biopsy
34	2	Negative	-1.2	Concordant	90	3.9	Concordant	60	1.8	Concordant	14	-3.7	Discordant	Core biopsy
35	2	Negative	-0.7	Discordant	95	4.4	Concordant	80	1.3	Concordant	12	-4.4	Concordant	Core biopsy
36	2	Negative	-1.1	Concordant	95	3.4	Concordant	0	-1.4	Discordant	5	-3.7	Discordant	Core biopsy
37	2	Negative	-1.2	Concordant	90	4	Concordant	0	-6.2	Concordant	13	-3.3	Discordant	Core biopsy
39	2	Negative	-1.9	Concordant	0	-1.7	Concordant	0	-1.9	Discordant	12	-3.2	Discordant	Core biopsy
40	2	Negative	-3.3	Concordant	90	0.9	Concordant	0	-2.8	Discordant	12	-4.6	Concordant	Core biopsy
43	2	Negative	-1.9	Concordant	100	4.1	Concordant	70	0.9	Concordant	15	-1.1	Discordant	Core biopsy
44	2	Negative	-3.1	Concordant	90	1.7	Concordant	70	1.1	Concordant	5	-2.5	Discordant	Core biopsy
45	2	Negative	-0.7	Discordant	80	1.8	Concordant	80	0.6	Concordant	50	-1.9	Concordant	Core biopsy
46	2	Negative	-1.5	Concordant	100	3.1	Concordant	15	-1.8	Concordant	8	-3.4	Discordant	Core biopsy
48	2	Negative	0.1	Discordant	100	4.4	Concordant	60	-8.8	Discordant	12	-3.4	Discordant	Core biopsy
49	2	Negative	-1.1	Concordant	90	3.8	Concordant	80	1.1	Concordant	17	-2.3	Discordant	Core biopsy
50	2	Negative	-2.4	Concordant	100	3.4	Concordant	15	-0.5	Concordant	18	-1.4	Discordant	Core biopsy
52	2	Negative	-4.6	Concordant	0	0.7	Discordant	0	-1.2	Discordant	28	-6.6	Discordant	Core biopsy
54	2	Positive	-2	Discordant	0	-2.4	Concordant	0	-3.9	Concordant	25	-1	Concordant	Core biopsy
55	2	Positive	0.7	Concordant	0	-10	Concordant	0	-8.8	Concordant	17	-1.1	Discordant	Core biopsy
56	2	Positive	2.3	Concordant	70	0.2	Concordant	0	-1.7	Discordant	30	-1.9	Concordant	Core biopsy
57	2	Positive	1.9	Concordant	90	2.2	Concordant	90	1.5	Concordant	20	-2.1	Discordant	Core biopsy
59	2	Positive	-3.7	Discordant	75	2.5	Concordant	90	2.2	Concordant	20	-1	Discordant	Core biopsy
60	2	Positive	0	Concordant	70	1	Concordant	50	-0.4	Concordant	12	-2.9	Discordant	Core biopsy
61	2	Positive	-1.4	Discordant	90	2	Concordant	5	-3	Concordant	13	-1.7	Discordant	Core biopsy
62	2	Positive	-2	Discordant	90	2.3	Concordant	15	-2.7	Concordant	25	-1.9	Concordant	Core biopsy
63	2	Positive	-0.1	Concordant	40	3	Concordant	30	-1.1	Concordant	60	-10	Discordant	Core biopsy

64	2	Negative	-1.3	Concordant	70	1	Concordant	70	0.9	Concordant	20	-1.4	Discordant	Core biopsy
65	2	Negative	-0.9	Discordant	100	4	Concordant	35	0.5	Concordant	12	-3.9	Discordant	Core biopsy
66	2	Negative	-1.3	Concordant	70	3.1	Concordant	0	-2.4	Discordant	15	-1.9	Discordant	Core biopsy
71	2	Negative	-3	Concordant	80	2	Concordant	15	-1.8	Concordant	10	-2.7	Discordant	Core biopsy
72	2	Negative	-1	Discordant	100	4.7	Concordant	80	-0.7	Concordant	10	-4.2	Concordant	Core biopsy
74	2	Negative	-4	Concordant	0	-0.9	Discordant	0	-5.6	Concordant	45	0.7	Concordant	Core biopsy
83	2	Negative	-1.5	Concordant	0	-4.5	Concordant	0	-3.4	Discordant	22	-1.8	Concordant	Core biopsy
87	2	Negative	-1	Discordant	0	-3.4	Concordant	0	-5.1	Concordant	15	-2.5	Discordant	Core biopsy
109	2	Negative	-2.3	Concordant	80	3.8	Concordant	70	1.9	Concordant	20	-2.5	Discordant	Surgical specimen
135	2	Negative	-3.6	Concordant	80	4.8	Concordant	30	1.8	Concordant	10	-2.1	Discordant	Surgical specimen
143	2	Negative	-1.7	Concordant	100	3.5	Concordant	0.5	-3.6	Concordant	15	-2	Discordant	Core biopsy
145	2	Negative	-1	Discordant	100	3.5	Concordant	60	-0.5	Concordant	2	-10	Concordant	Core biopsy
147	2	Negative	0.4	Discordant	100	3.6	Concordant	60	1.8	Concordant	5	-0.9	Discordant	Core biopsy
149	2	Negative	-2.9	Concordant	20	1.1	Concordant	2	0.3	Concordant	3	-3.7	Discordant	Core biopsy
170	2	Negative	-2.9	Concordant	95	2.9	Concordant	25	0	Concordant	7	-3.1	Discordant	Core biopsy
174	2	Negative	-1.1	Concordant	0	-3	Concordant	0	-4.1	Concordant	4	-4	Discordant	Core biopsy
190	2	Negative	-2.9	Concordant	80	2.2	Concordant	70	2.3	Concordant	10	-3.1	Discordant	Core biopsy
196	2	Negative	-2.2	Concordant	60	0.2	Concordant	0	-4.8	Concordant	20	-2.4	Discordant	Core biopsy
211	2	Negative	-2.9	Concordant	80	2.6	Concordant	80	2.7	Concordant	20	-3.3	Discordant	Core biopsy
226	2	Negative	-4.3	Concordant	80	1.6	Concordant	80	-1.4	Concordant	3	-3.9	Discordant	Core biopsy
228	2	Negative	-3.3	Concordant	100	4	Concordant	35	1.2	Concordant	17	-1.4	Discordant	Core biopsy
239	2	Negative	-2.8	Concordant	80	2.3	Concordant	20	0.9	Concordant	19	-2.1	Discordant	Core biopsy
243	2	Negative	-2.3	Concordant	70	0.8	Concordant	60	0.6	Concordant	10	-3.5	Discordant	Core biopsy
246	2	Negative	1.3	Discordant	80	2.1	Concordant	30	0	Concordant	20	-1.4	Discordant	Core biopsy
249	2	Negative	-3.1	Concordant	80	2.1	Concordant	60	1.3	Concordant	5	-3.5	Discordant	Core biopsy
252	2	Negative	-2.7	Concordant	95	3.5	Concordant	0	-7.3	Concordant	13	-3.9	Discordant	Core biopsy
77	0	Negative	-4.3	Concordant	0	-1.1	Concordant	0	-2.2	Discordant	3	-6.3	Concordant	Core biopsy

78	0	Negative	-2.3	Concordant	0	-4.9	Concordant	0	-1.8	Discordant	38	-0.3	Concordant	Core biopsy
81	0	Negative	-4.9	Concordant	0	-1.3	Concordant	0	-1.4	Discordant	5	-2.7	Discordant	Core biopsy
82	0	Negative	-3.1	Concordant	0	-2.3	Concordant	0	-1.1	Discordant	32	-0.7	Concordant	Core biopsy
85	0	Negative	-3.9	Concordant	0	-3.2	Concordant	0	-4.5	Concordant	10	-2	Discordant	Core biopsy
90	0	Negative	-2.7	Concordant	0	-2.2	Concordant	0	-2.7	Discordant	28	-1.9	Concordant	Core biopsy
92	0	Negative	-4	Concordant	80	1.2	Concordant	80	-1.6	Concordant	7	-2.2	Discordant	Surgical specimen
94	0	Negative	-2.2	Concordant	90	2.8	Concordant	40	-0.3	Concordant	20	-2.7	Discordant	Surgical specimen
96	0	Negative	-2.8	Concordant	90	3.9	Concordant	0	-3.4	Discordant	30	-2.2	Concordant	Surgical specimen
97	0	Negative	-3.6	Concordant	50	1	Concordant	50	-1.7	Concordant	16	-2.7	Discordant	Surgical specimen
100	0	Negative	-1.4	Concordant	60	2.1	Concordant	90	2.5	Concordant	15	-1.4	Discordant	Surgical specimen
101	0	Negative	-2.4	Concordant	90	4.6	Concordant	50	1.3	Concordant	15	-3.7	Discordant	Surgical specimen
102	0	Negative	-2.4	Concordant	50	1.6	Concordant	90	1.3	Concordant	15	-2.8	Discordant	Surgical specimen
104	0	Negative	-4	Concordant	90	1.5	Concordant	10	1.3	Concordant	20	-3	Discordant	Surgical specimen
106	0	Negative	-4.1	Concordant	50	1.7	Concordant	75	0.7	Concordant	20	-3	Discordant	Surgical specimen
107	0	Negative	-3.7	Concordant	90	2.5	Concordant	40	-3.3	Concordant	10	-2.3	Discordant	Surgical specimen
112	0	Negative	-2.4	Concordant	80	3.2	Concordant	0	-2	Discordant	15	-3.4	Discordant	Surgical specimen
113	0	Negative	-3.8	Concordant	80	2.2	Concordant	80	1.7	Concordant	5	-3.2	Discordant	Surgical specimen
114	0	Negative	-2.5	Concordant	60	1.1	Concordant	80	-0.8	Concordant	10	-4	Discordant	Surgical specimen
120	0	Negative	-5.1	Concordant	90	-1.8	Discordant	20	-4	Discordant	15	-5.9	Concordant	Surgical specimen
124	1	Negative	-2.2	Concordant	60	2.1	Concordant	80	-1.9	Concordant	20	-2.6	Discordant	Surgical specimen

125	0	Negative	-2.4	Concordant	70	2.3	Concordant	0	-1	Discordant	15	-5.4	Concordant	Surgical specimen
126	0	Negative	-7.1	Concordant	90	2.7	Concordant	70	2.7	Concordant	16	-2.7	Discordant	Surgical specimen
129	0	Negative	-5.4	Concordant	80	-0.8	Concordant	60	-1	Concordant	50	-4.7	Discordant	Surgical specimen
132	0	Negative	-2.5	Concordant	60	3.2	Concordant	0	-0.4	Discordant	10	-4.2	Concordant	Surgical specimen
133	0	Negative	-2.2	Concordant	80	4.9	Concordant	25	1.1	Concordant	15	-0.6	Discordant	Surgical specimen
136	0	Negative	-2.2	Concordant	70	2.9	Concordant	30	0.7	Concordant	10	-1.6	Discordant	Surgical specimen
138	0	Negative	-2.6	Concordant	90	1.6	Concordant	70	1.7	Concordant	15	-3.5	Discordant	Surgical specimen
144	0	Negative	-1.4	Concordant	0	-7.1	Concordant	0	-5.9	Concordant	NA	-0.7	Discordant	Core biopsy
148	1	Negative	-3.8	Concordant	70	2.9	Concordant	0	-1.8	Discordant	2	-3.9	Discordant	Core biopsy
150	0	Negative	-3.5	Concordant	90	3.6	Concordant	90	2.3	Concordant	2	-4	Discordant	Core biopsy
153	1	Negative	-4.1	Concordant	90	2	Concordant	0	-3.5	Discordant	20	-0.8	Discordant	Core biopsy
155	0	Negative	-3.4	Concordant	12	-0.2	Concordant	60	-0.5	Concordant	7	-2.5	Discordant	Core biopsy
156	1	Negative	-2.6	Concordant	80	2.9	Concordant	80	2.1	Concordant	20	-1.9	Discordant	Core biopsy
163	0	Negative	-3.7	Concordant	40	1.8	Concordant	0	-1.5	Discordant	10	-4.2	Concordant	Core biopsy
165	1	Negative	-3.1	Concordant	10	1.4	Concordant	70	2.6	Concordant	12	-3	Discordant	Core biopsy
172	0	Negative	-5.1	Concordant	95	3.5	Concordant	40	2	Concordant	12	-2.1	Discordant	Core biopsy
173	1	Negative	-3.6	Concordant	70	1.4	Concordant	0	-2.1	Discordant	5	-10	Concordant	Vacuum assited biopsy
176	1	Negative	-3.9	Concordant	90	4.1	Concordant	90	0.6	Concordant	5	-3.2	Discordant	Core biopsy
180	1	Negative	-3	Concordant	90	3.2	Concordant	0	-2.1	Discordant	3	-3.9	Discordant	Core biopsy
181	1	Negative	-2.1	Concordant	95	3.6	Concordant	70	0.4	Concordant	15	-3.1	Discordant	Core biopsy
182	1	Negative	-1.8	Concordant	90	4	Concordant	0	-2.8	Discordant	5	-6.4	Concordant	Core biopsy
183	0	Negative	-5.3	Concordant	80	3.2	Concordant	0	1.4	Discordant	20	-1.9	Discordant	Core biopsy
184	1	Negative	-3.6	Concordant	60	2.6	Concordant	0	-3.5	Discordant	5	-3.7	Discordant	Core biopsy
187	1	Negative	-4.2	Concordant	90	3.8	Concordant	1	-3.9	Discordant	2	-4.3	Concordant	Core biopsy

188	0	Negative	-3.1	Concordant	70	2.1	Concordant	20	0	Concordant	20	-2	Discordant	Core biopsy
191	0	Negative	-3.5	Concordant	40	2.4	Concordant	0	-0.4	Discordant	5	-4.2	Concordant	Core biopsy
194	1	Negative	-3.5	Concordant	70	2.2	Concordant	70	1.4	Concordant	10	-3.2	Discordant	Core biopsy
195	0	Negative	-1.4	Concordant	90	5.3	Concordant	2	-2	Concordant	10	-2.5	Discordant	Core biopsy
208	1	Negative	-2.1	Concordant	100	4.4	Concordant	50	0.9	Concordant	18	-1.5	Discordant	Core biopsy
209	1	Negative	-2	Concordant	65	5	Concordant	0	2.1	Discordant	3	-3.6	Discordant	Core biopsy
222	1	Negative	-2.5	Concordant	80	3	Concordant	20	0.3	Concordant	10	-2.4	Discordant	Core biopsy
225	1	Negative	-2.4	Concordant	80	1.6	Concordant	40	0.1	Concordant	5	-3.9	Discordant	Core biopsy
238	1	Negative	-4.6	Concordant	80	4.7	Concordant	0	-8.6	Concordant	5	-3	Discordant	Core biopsy
244	0	Negative	-2.7	Concordant	100	4.5	Concordant	100	2	Concordant	5	-3.1	Discordant	Core biopsy
245	0	Negative	-3.9	Concordant	0	-10	Concordant	8	-5.7	Discordant	80	0.5	Concordant	Core biopsy
247	1	Negative	-3	Concordant	90	2.7	Concordant	60	0.3	Concordant	20	-2.9	Discordant	Core biopsy
253	3	Positive	1.4	Concordant	0	-8.1	Concordant	0	-5.2	Concordant	17	-1.4	Discordant	core biopsy
258	3	Positive	1.6	Concordant	70	0.1	Concordant	100	1.5	Concordant	8	-3.2	Discordant	Core biopsy
259	3	Positive	0.9	Concordant	0	-2.9	Concordant	0	-4.3	Concordant	12	-3	Discordant	Core biopsy
260	3	Positive	2.1	Concordant	5	-1.5	Discordant	5	-2.3	Concordant	12	-2.8	Discordant	Core biopsy
261	3	Positive	0.6	Concordant	90	1.2	Concordant	50	-1	Concordant	18	-2.3	Discordant	Core biopsy
262	3	Positive	3.2	Concordant	0	-2.9	Concordant	0	-3.1	Discordant	50	-1.8	Concordant	Core biopsy
263	3	Positive	0.8	Concordant	0	-3.5	Concordant	0	-3.1	Discordant	20	-2	Discordant	Core biopsy
265	3	Positive	0.4	Concordant	70	1.1	Concordant	60	0	Concordant	18	-2.7	Discordant	Core biopsy
267	3	Positive	1.6	Concordant	60	0.5	Concordant	0	-5.3	Concordant	17	-3.4	Discordant	Core biopsy
268	3	Positive	1.2	Concordant	0	-1.1	Concordant	0	-10	Concordant	9	-1.8	Discordant	Surgical specimen
269	3	Positive	2.5	Concordant	90	2.9	Concordant	20	-0.7	Concordant	18	-2.9	Discordant	Core biopsy
273	3	Positive	2.6	Concordant	10	-1.6	Discordant	0	-10	Concordant	50	-1.2	Concordant	Core biopsy
274	3	Positive	1.4	Concordant	0	-2.9	Concordant	0	-4.9	Concordant	18	-1.4	Discordant	Core biopsy
275	3	Positive	1.8	Concordant	0	-6.4	Concordant	0	-10	Concordant	19	-1.6	Discordant	Core biopsy
279	3	Positive	-1.1	Discordant	90	1.8	Concordant	100	1.5	Concordant	40	-0.8	Concordant	Core biopsy
280	3	Positive	1	Concordant	80	2.9	Concordant	5	-8.2	Discordant	28	-1.5	Concordant	Core biopsy
